# TMEM16F exacerbates tau pathology and mediates phosphatidylserine exposure in phospho-tau-burdened neurons

**DOI:** 10.1073/pnas.2311831121

**Published:** 2024-06-28

**Authors:** Mario V. Zubia, Adeline J. H. Yong, Kristen M. Holtz, Eric J. Huang, Yuh Nung Jan, Lily Y. Jan

**Affiliations:** ^a^Biomedical Sciences Graduate Program, University of California, San Francisco, CA 94143; ^b^Department of Physiology, University of California, San Francisco, CA 94143; ^c^Department of Biochemistry and Biophysics, University of California, San Francisco, CA 94143; ^d^HHMI, University of California, San Francisco, CA 94143; ^e^Department of Autonomy, Skydio, San Mateo, CA 94402; ^f^Department of Pathology, University of California, San Francisco, CA 94143

**Keywords:** TMEM16F, P301S (PS19), tauopathy, lipid scrambling, phosphatidylserine exposure

## Abstract

TMEM16F is a calcium-activated phospholipid scramblase and nonselective ion channel, which allows the movement of lipids bidirectionally across the plasma membrane. While the functions of TMEM16F have been extensively characterized in multiple cell types, the role of TMEM16F in the central nervous system remains largely unknown. Here, we sought to study how TMEM16F in the brain may be involved in neurodegeneration. Using a mouse model that expresses the pathological P301S human tau (PS19 mouse), we found reduced tauopathy and microgliosis in 6- to 7-mo-old PS19 mice lacking TMEM16F. Furthermore, this reduction of pathology can be recapitulated in the PS19 mice with TMEM16F removed from neurons, while removal of TMEM16F from microglia of PS19 mice did not significantly impact tauopathy at this time point. Moreover, TMEM16F mediated aberrant phosphatidylserine exposure in neurons with phospho-tau burden. These studies raise the prospect of targeting TMEM16F in neurons as a potential treatment of neurodegeneration.

## Significance

TMEM16F is widely expressed across the body, but its role in the brain and neurodegeneration is largely unexplored. This study provides evidence of neuronal TMEM16F-mediated aberrant phosphatidylserine exposure in early-stage tauopathy and neurodegeneration. Our results suggest that reducing TMEM16F activity, specifically in neurons, has potential therapeutic implication in mitigating tauopathy.

TMEM16F, also known as anoctomin-6 (Ano6), has the dual function of ion channel and phospholipid scramblase (1, 2). Lipids are asymmetrically distributed across the phospholipid bilayer, with phosphatidylcholine and various sphingolipids on the outer leaflet, and phosphatidylserine, phosphatidylethanolamine, and phosphatidylinositols on the inner leaflet of the plasma membrane (3, 4). This distribution is established and maintained through ATP-dependent translocases referred to as “flippases,” which move lipids from the outer to inner leaflet, and “floppases,” which move lipids from the inner to outer leaflet (3). A third class, known as lipid “scramblases,” allows for the movement of lipids bidirectionally, in a Ca^2+^-dependent, ATP-independent manner, leading to the exposure of phosphatidylserine to the cell surface (3).

Asymmetric distribution of lipids plays an important role in numerous cellular functions including protein docking, blood coagulation, and formation of various intra- and extracellular vesicles (EVs) (4, 5). In blood coagulation, activated platelets externalize negatively charged phosphatidylserine, which serves as a scaffold for tissue factors in the coagulation cascade (4, 5). Clinically, patients with a bleeding disorder called Scott syndrome have coagulation deficits due to impaired lipid scrambling on activated platelets (6). This impairment was found to be mediated by loss of function mutations of TMEM16F (7). Following the discovery of the critical role of TMEM16F in blood coagulation, numerous studies have examined this lipid scrambling role of TMEM16F (8, 9). Its function as a Ca^2+^-activated nonselective ion channel has been shown to mediate calcium influx, which can provide a positive feedback mechanism to enhance the Ca^2+^-activated phospholipid scramblase activity (10). The mechanism by which scrambling occurs is currently being investigated, to examine whether TMEM16F employs separate pathways for ion permeation and phospholipid scrambling (11–17).

While TMEM16F is expressed in most if not all cells throughout the body, most studies have focused on immune cells, including platelets (18, 19), B cells (8), T cells (20, 21), and neutrophils (22). The role of TMEM16F in the nervous system is still largely unknown. In microglia, the resident immune cells in the brain and spinal cord, TMEM16F facilitates microglial dysfunction in neuropathic pain states (23), in inflammatory polarization following spinal cord injury (24), and in Alzheimer’s disease (25). In neurons, TMEM16F may contribute to cholinergic regulation of motoneurons (26) and phosphatidylserine-mediated phagocytosis of neurons after cerebral ischemia (27).

In this study, we sought to explore the role of TMEM16F in neurodegeneration in the P301S tau (PS19) mouse model of tauopathy, which expresses mutant human tau with the P301S mutation found in frontotemporal dementia and Parkinsonism linked to chromosome 17 (FTDP-17) patients (28). Neurodegenerative disease is characterized by the progressive degeneration of neurons, in their structure and connectivity or in their function, leading to cell death (29). There are many types of neurodegenerative disorders, with one of the most prominent being Alzheimer’s disease, which accounts for 60 to 80% of all cases of dementia (29, 30). Alzheimer’s disease affects 24 million people worldwide, is the sixth leading cause of death, and has placed a several hundred-billion-dollar burden on the healthcare system for both patients and their caretakers (30). The pathology of Alzheimer’s disease is characterized as the buildup of extracellular amyloid beta (Aβ) plaques as well as intracellular neurofibrillary tau tangles that are composed of hyperphosphorylated tau filaments and tau aggregates (29). Both proteins have been shown to induce deficits and degeneration (29). However, in patients, the rate of pathogenic buildup of tau, rather than Aβ, is associated with cognitive deficits (31). Furthermore, while Alzheimer’s disease is the most common dementia, neurofibrillary tau tangles are the most common intracellular inclusion found in over twenty-five types of neurodegenerative disorders as either primary or secondary tauopathies (29). Given that many mechanisms are shared across these diseases involving tauopathies, it can be beneficial to study tauopathies in mouse models of tauopathy.

By examining PS19 mice with complete deletion of TMEM16F or PS19 mice with conditional removal of TMEM16F from either neurons or microglia, we found that the presence of TMEM16F in neurons significantly worsened tau pathology in hippocampal neurons. We further validated these results using primary hippocampal neuron cultures to reveal that phospho-tau-burdened neurons aberrantly expose phosphatidylserine through TMEM16F.

## Results

### Loss of TMEM16F Reduced Tau Pathology.

**Fig. 1.**
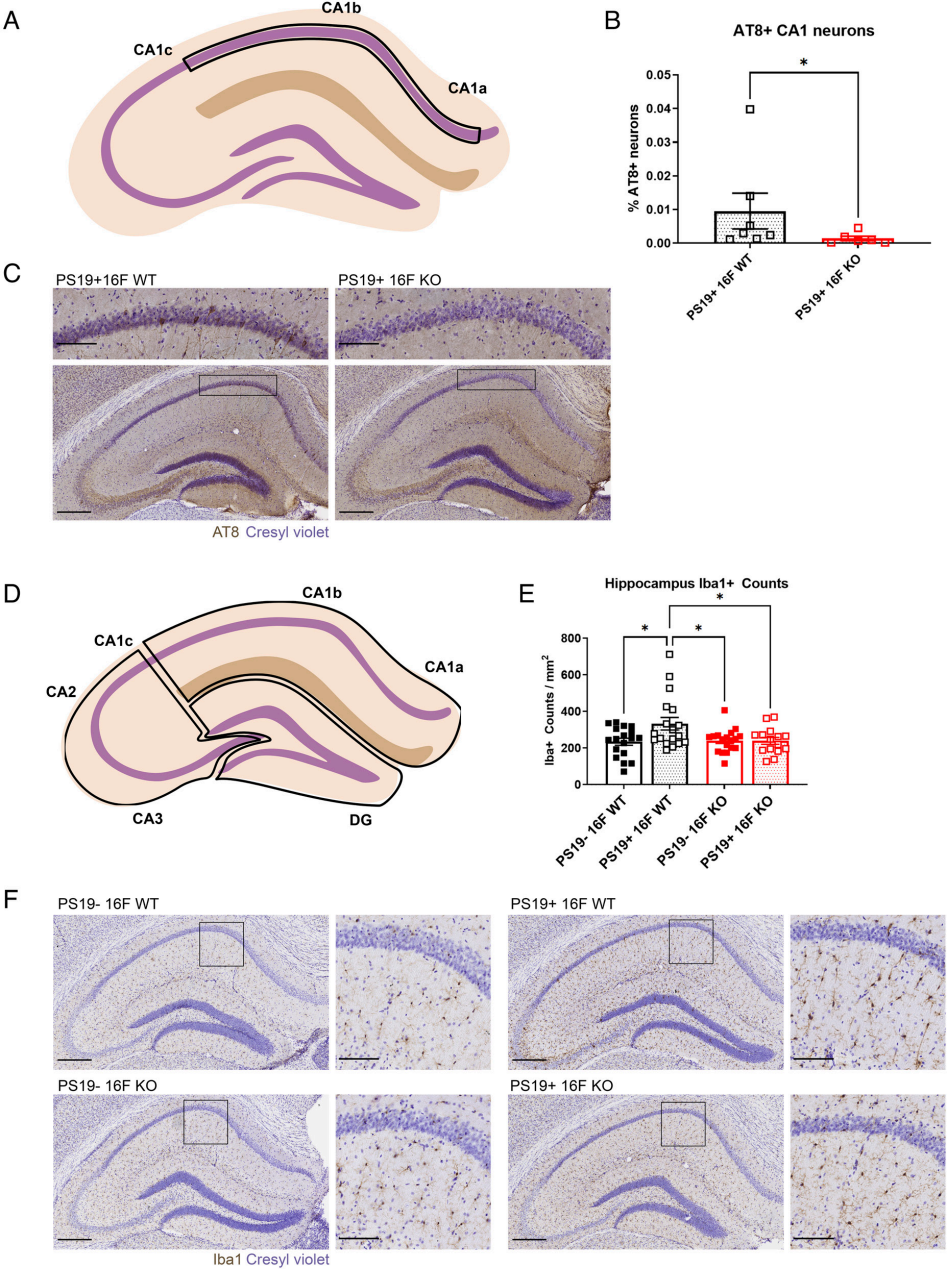
TMEM16F KO mice with reduced tau pathology. (*A*) Depiction of CA1a-b region of interest (within the boxed region) used for AT8 analysis. (*B*) Quantification of AT8+ neurons within the CA1 pyramidal layer in PS19+ WT, n = 7 and PS19+ KO mice, n = 6; 3 to 4 hippocampal sections/mouse. “% AT8+ neurons” gives number of AT8+ neurons normalized by area of interest and total number of neurons per area (neuronal density in the CA1 pyramidal layer) (*P* = 0.035, Mann–Whitney test). Error bars in SEM. (*C*) Representative images of AT8 immunostaining of hyperphosphorylated tau in the hippocampus and CA1 (*Inset*) in 6-mo-old PS19+ TMEM16F WT (16F WT) and TMEM16F KO (16F KO) mice. Cresyl violet, neuronal counterstain. (Scale bar, 300 µm for hippocampus and 100 µm for *Inset*.) (*D*) Depiction of CA1 (CA1a-b), CA3 (CA1c, CA2, CA3), and DG regions of interest (ROIs) (within the three boxed regions) used for Iba1 analysis. (*E*) Quantification of number of microglia per area (count/mm^2^) in the hippocampus of 6-mo-old PS19− 16F WT, PS19− 16F KO, PS19+ 16F WT, and PS19+ 16F KO mice, n = 6 per genotype, three ROIs per mouse (*P < 0.05, two-way ANOVA, Tukey’s multiple comparisons test). Error bars in SEM. (*F*) Representative images of Iba1 immunostaining of microglia in the hippocampus and CA1 (*Inset*) of 6-mo-old PS19− 16F WT, PS19− 16F KO, PS19+ 16F WT, and PS19+ 16F KO mice. Cresyl violet, neuronal counterstain. (Scale bar, 300 µm for hippocampus and 100 µm for *Inset*.)

To assess how TMEM16F affects tauopathy, we crossed TMEM16F knockout (KO) mice with PS19 mice to generate PS19+ TMEM16F WT (PS19+ 16F WT) and PS19+ TMEM16F KO (PS19+ 16F KO) mice. Assessment of PS19+ 16F WT and PS19+ 16F KO mice at 3 mo of age showed no hyperphosphorylated tau (AT8 staining) in hippocampal neurons in either genotype (*SI Appendix*, Fig. S1). This is consistent with previous studies of PS19 mice (28, 32) and suggests that complete KO of TMEM16F does not exacerbate disease progression in young mice (28). Staining pattern and progression of hyperphosphorylated tau throughout the hippocampus in the PS19 model has been previously reported (33, 34). Mice with the lowest disease severity begin exhibiting AT8 positivity in the mossy fibers of CA3 and hilus of the dentate gyrus (DG), with gradual expansion to CA1 neurons and their neurites, before widespread appearance of hyperphosphorylated tau throughout the entire hippocampus. In our study of 6- to 7-mo-old mice, AT8 positivity did not extend past sparse CA1 staining and thus for quantification, we counted CA1a-b (simplified as CA1, henceforth) pyramidal neurons with strong AT8+ signal (region demonstrated in Fig. 1*A*). At 6 mo of age, PS19+ 16F WT mice have significantly more hyperphosphorylated tau within CA1 pyramidal neurons compared to their PS19+ TMEM16F KO (PS19+ 16F KO) littermates (Fig. 1 *B* and *C*). While not all PS19+ 16F WT mice displayed this robust AT8+ staining, those that did had many more AT8+ neurons within this region (Fig. 1*C*).

Since previous studies have shown that tauopathy in the hippocampus of PS19 mice induces robust microglial responses (33), we asked whether PS19+ 16F KO also affected the microgliosis phenotype. Within the brain, microglia have distinct territories in which they surveil nearby neurons (35). In response to neurodegeneration, microglia often proliferate, thereby increasing microglial density, and neuronal damage is generally exacerbated (36). Using microglial density as an indicator of microgliosis, we measured counts from three regions (Fig. 1*D*) and found more numerous hippocampal microglia (marked with Iba1 staining) in PS19+ 16F WT mice compared to those in nontauopathy, control PS19− 16F WT mice (Fig. 1 *E* and *F*). KO of TMEM16F in tauopathy mice restored the microglial density to that of control mice (Fig. 1 *E* and *F*). These data suggest that TMEM16F in this PS19 mouse model contributes to tau pathology.

### Testing the Function of Microglial TMEM16F in Tau Pathology.

Having found that removal of TMEM16F reduced the severity of tauopathy in hippocampal neurons and microgliosis in PS19+ 16F KO mice, we next sought to determine which cells may account for this effect. Several different cell types have been implicated in tau spreading and advancement of pathology (37). Phospho-tau seeds from neurons can be translocated to other neurons or secreted into the extracellular space, either directly or through EVs (37–39). Transfer of tau via EVs may also involve microglia (40, 41), which phagocytose dying neurons and can be activated by various molecules that are released into the extracellular space (35). In tauopathy, the failure of microglia to fully phagocytose and degrade tau aggregates could release tau oligomers and facilitate prion-like spread to neurons (40). Because removal of TMEM16F in microglia has been shown to be protective in neuropathic pain states (23), spinal cord injury (24), and Alzheimer’s disease (25), we asked whether TMEM16F removal from microglia can mitigate tauopathy.

**Fig. 2.**
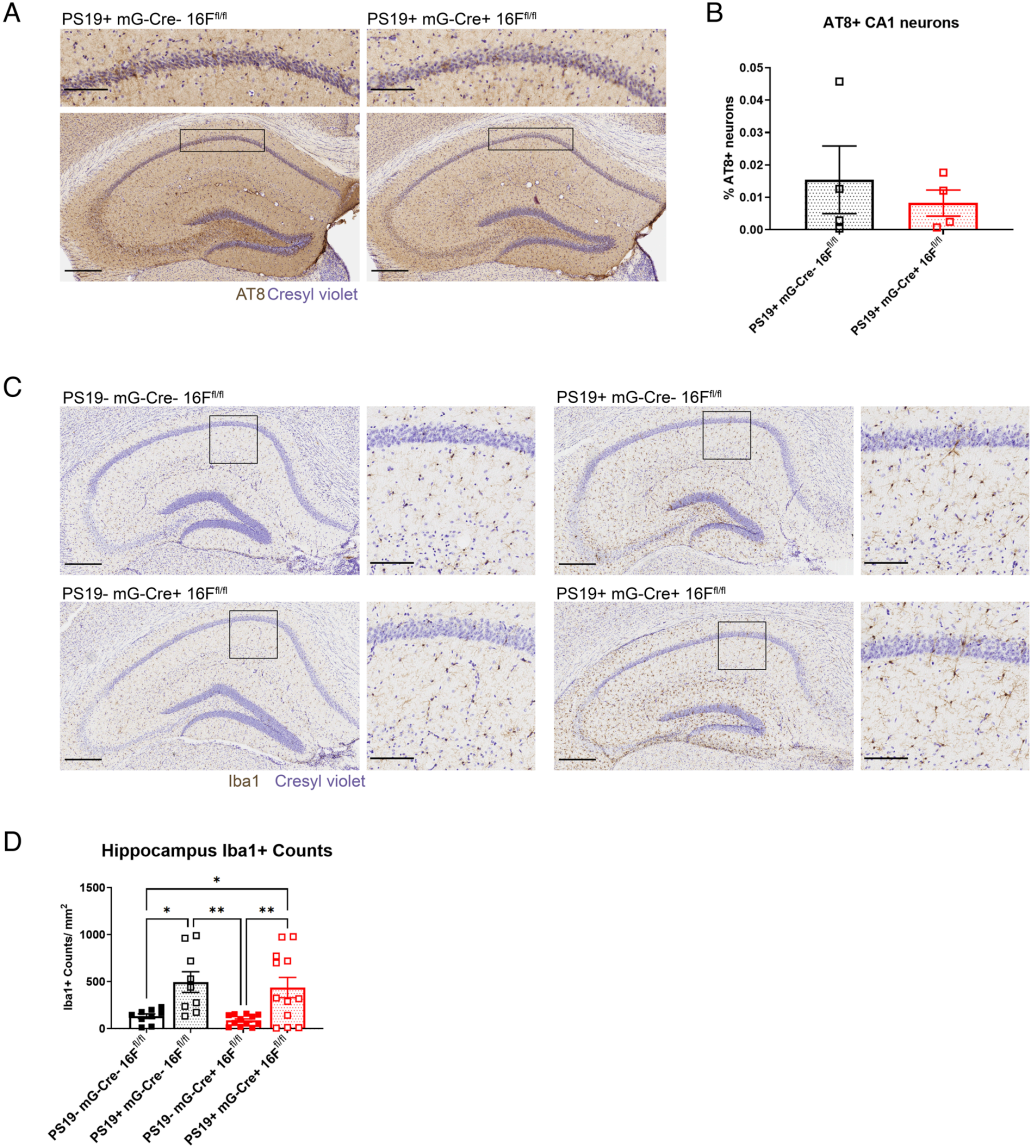
Microglial TMEM16F conditional KO mice without detectable effect on disease pathology. (*A*) Representative images of AT8 immunostaining of hyperphosphorylated tau in the hippocampus and CA1 (*Inset*) in 7-mo-old PS19+ microglial TMEM16F WT (mG-Cre− 16F^fl/fl^) and microglial TMEM16F conditional KO mice (mG-Cre+ 16F^fl/fl^). Cresyl violet, neuronal counterstain. (Scale bar, 300 µm for hippocampus and 100 µm for *Inset*.) (*B*) Quantification of AT8+ neurons within CA1 pyramidal layer in PS19+ mG-Cre− 16F^fl/fl^, n = 4 and PS19+ mG-Cre+ 16F^fl/fl^ mice, n = 6; 3 to 4 hippocampal sections/ mouse. % AT8+ neurons gives number of AT8+ neurons normalized by area of interest and total number of neurons per area (neuronal density in the CA1 pyramidal layer) (*P* = 0.8857, Mann–Whitney test). Error bars in SEM. (*C*) Representative images of Iba1 immunostaining of microglia in the hippocampus and CA1 (*Inset*) of 7-mo-old PS19− mG-Cre− 16F^fl/fl^, PS19− mG-Cre+ 16F^fl/fl^, PS19+ mG-Cre− 16F^fl/fl^, and PS19+ mG-Cre+ 16F^fl/fl^ mice. Cresyl violet, neuronal counterstain. (Scale bar, 300 µm for hippocampus and 100 µm for *Inset*.) (*D*) Quantification of number of microglia per area (count/mm^2^) in the hippocampus of 7-mo-old PS19− mG-Cre− 16F^fl/fl^, PS19− mG-Cre+ 16F^fl/fl^, PS19+ mG-Cre− 16F^fl/fl^, and PS19+ mG-Cre+ 16F^fl/fl^ mice, n = 4 to 5 per genotype, three ROIs per mouse (*P < 0.05, **P < 0.01, two-way ANOVA, Tukey’s multiple comparisons test). Error bars in SEM.

To this end, we removed TMEM16F from microglia by crossing Cx3cr1-Cre TMEM16F flox/flox (mG-Cre+ 16F^fl/fl^) mice with PS19 mice (42). Under this breeding scheme, PS19+ mG-Cre− 16F^fl/fl^ mice did not exhibit significant differences in AT8+ staining or microgliosis compared to control PS19− 16F mG-Cre− 16F^fl/fl^ mice at 6 mo of age (*SI Appendix*, Fig. S2). Given that several studies have reported a similar delay in onset in mice with mixed genetic background (32, 43, 44), we decided to test 7-mo-old mice. In 7-mo-old mice, we detected tau pathology and microgliosis in tauopathy PS19+ mG-Cre− 16F^fl/fl^ mice but not in control PS19− mG-Cre− 16F^fl/fl^ mice (Fig. 2 *A*, *B*, and *D*). Thus, we selected this time point for analyses of conditional KO mice. There was no significant difference in the number of neurons with hyperphosphorylated tau in CA1 of PS19+ mG-Cre− 16F^fl/fl^ mice compared to those with TMEM16F conditionally knocked out in microglia (PS19+ mG-Cre+ 16F^fl/fl^) (Fig. 2 *A* and *B*). We also found that removal of microglial TMEM16F did not significantly reduce microglial density in PS19+ mG-Cre+ 16F^fl/fl^ mice compared to that in PS19+ mG-Cre− 16F^fl/fl^ mice (Fig. 2 *C* and *D*). It thus appears that microglial TMEM16F does not significantly contribute to tauopathy at this 6- to 7-mo timepoint.

### TMEM16F Function in Neurons Is Important for Tau Pathology.

Having seen no significant effect of TMEM16F removal from microglia on tauopathy pathology, we next tested for TMEM16F function in neurons. In tauopathy, neurons are both carriers and propagators of tau and hyperphosphorylated tau (45, 46). Physiologically, tau binds and stabilizes microtubules within axons (46, 47). Phosphorylation or other posttranslational modifications of tau along its microtubule binding domain destabilizes its binding and promotes self-assembly into oligomers and higher-order fibrils (45, 46). Mislocalization and formation of these aggregates of tau gradually overburden neurons and affect their cellular function, thereby leading to neuronal death (46).

**Fig. 3.**
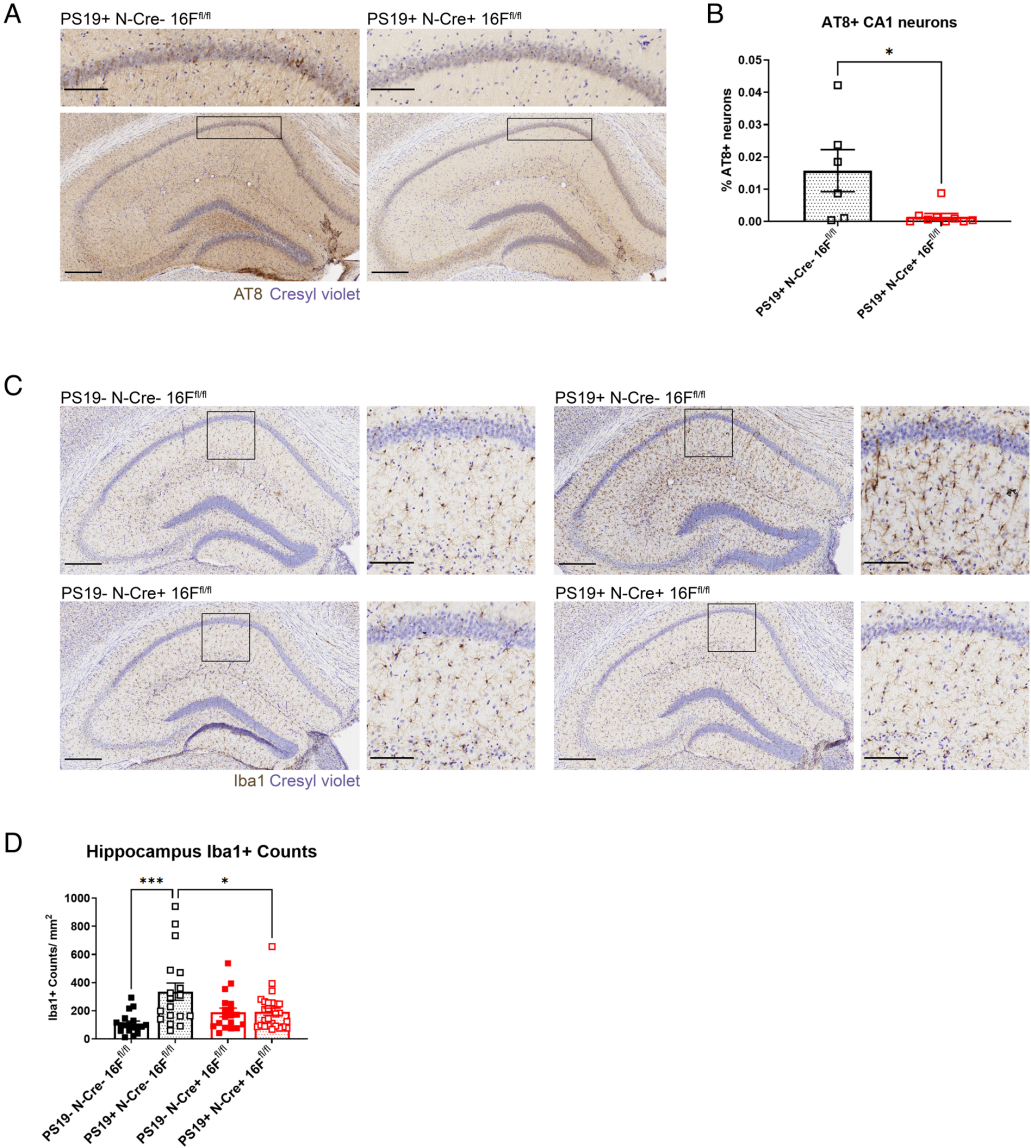
Removal of TMEM16F from neurons reduces disease pathology. (*A*) Representative images of AT8 immunostaining of hyperphosphorylated tau in the hippocampus and CA1 (*Inset*) in 7-mo-old PS19+ neuronal TMEM16F WT (N-Cre− 16F^fl/fl^) and neuronal TMEM16F conditional KO mice (N-Cre+ 16F^fl/fl^). Cresyl violet, neuronal counterstain. (Scale bar, 300 µm for hippocampus and 100 µm for *Inset*.) (*B*) Quantification of AT8+ neurons within CA1 pyramidal layer in PS19+ N-Cre− 16F^fl/fl^, n = 6, and PS19+ N-Cre+ 16F^fl/fl^ mice, n = 8; 3 to 4 hippocampal sections/mouse. % AT8+ neurons gives number of AT8+ neurons normalized by area of interest and total number of neurons per area (neuronal density in the CA1 pyramidal layer) (*P* = 0.04, Welch’s t test). Error bars in SEM. (*C*) Representative images of Iba1 immunostaining of microglia in the hippocampus and CA1 (*Inset*) of 7-mo-old PS19− N-Cre− 16F^fl/fl^, PS19− N-Cre+ 16F^fl/fl^, PS19+ N-Cre− 16F^fl/fl^, and PS19+ N-Cre+ 16F^fl/fl^ mice. Cresyl violet, neuronal counterstain. (Scale bar, 300 µm for hippocampus and 100 µm for *Inset*.) (*D*) Quantification of number of microglia per area (count/mm^2^) in the hippocampus of 7-mo-old PS19− N-Cre− 16F^fl/fl^, PS19− N-Cre+ 16F^fl/fl^, PS19+ N-Cre− 16F^fl/fl^, and PS19+ N-Cre+ 16F^fl/fl^ mice, n = 6 to 8 per genotype, three ROIs per mouse (*P < 0.05, ***P < 0.001, two-way ANOVA, Tukey’s multiple comparisons test). Error bars in SEM.

For conditional KO of TMEM16F in neurons, we chose the Baf53b (Actl6)-Cre line, as Baf53b (Actl6) is an RNA-binding protein with panneuronal expression (48). We crossed TMEM16F flox/flox mice with Baf53b-Cre (neuronal Cre, N-Cre) and PS19+ mice. As with the Cx3cr1-Cre mice, we assessed pathology at 7 mo of age because these mice shared the same background and likely had similar disease progression. In 7-mo-old mice, we found that tauopathy mice with TMEM16F conditionally knocked out from neurons under Baf53b-Cre (PS19+ N-Cre+ 16F^fl/fl^) had significantly fewer AT8+ neurons in CA1 compared with PS19+ N-Cre− 16F^fl/fl^ mice (Fig. 3 *A* and *B*), indicating that TMEM16F in neurons contributes to tau pathology. Likewise, when we assessed microgliosis, we found that removal of TMEM16F from neurons reduced levels of microgliosis in tauopathy mice (PS19+ N-Cre+ 16F^fl/fl^) compared to those with TMEM16F intact (PS19+ N-Cre− 16F^fl/fl^) (Fig. 3 *C* and *D*). These data suggest neuronal TMEM16F is affecting tauopathy pathology in both total number of CA1 neurons with hyperphosphorylated tau and in microglial response leading to increase of their density.

### Effect of TMEM16F Removal on Phosphatidylserine Exposure in Phospho-Tau-Burdened Neurons.

**Fig. 4.**
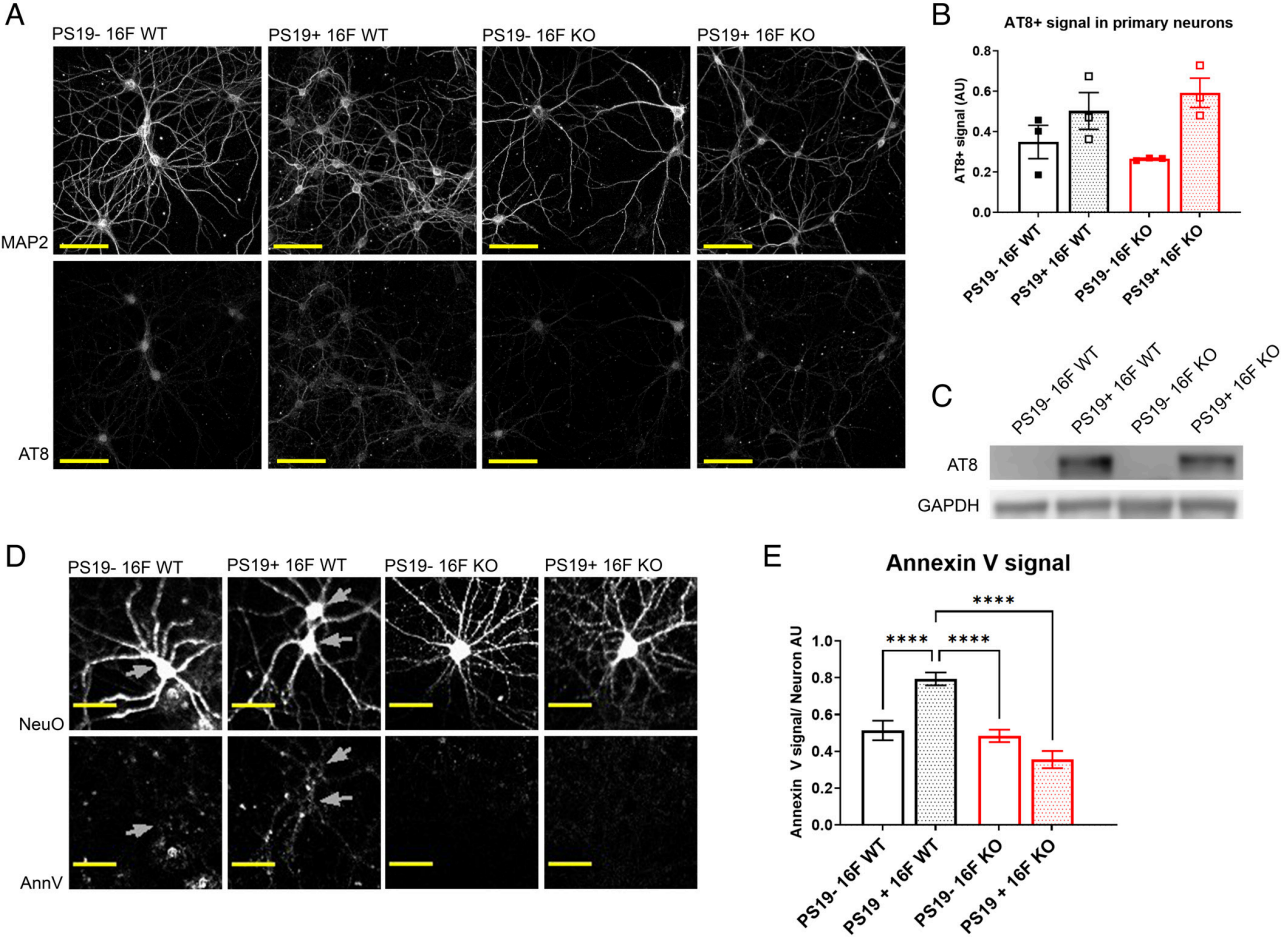
PS19+ neurons expose phosphatidylserine in a TMEM16F-dependent manner. (*A*) Representative images of NeuroFluor NeuO (neuronal marker) and AT8 (hyperphosphorylated tau) immunostaining in primary culture of PS19− TMEM16F WT (16F WT), PS19+ 16F WT, PS19− TMEM16F KO (16F KO), and PS19+ 16F KO hippocampal neurons. (*B*) Quantification of AT8 signals of primary PS19− 16F WT, PS19+ 16F WT, PS19− 16F KO, and PS19+ 16F KO neurons. n = 3, average of 20 fields per experiment. Error bars in SEM. (*C*) Representative Western blot of primary PS19− 16F WT, PS19+ 16F WT, PS19− 16F KO, and PS19+ 16F KO neuronal cell lysate probed with antibodies against AT8 and GAPDH for loading control. (*D*) Representative images of NeuroFluor NeuO (neuronal marker) and annexin V (exposed phosphatidylserine) immunostaining in primary cultures of PS19− 16F WT, PS19+ 16F WT, PS19− 16F KO, and PS19+ 16F KO hippocampal neurons. (Scale bar, 50 µm in all images.) (*E*) Quantification of average annexin V fluorescence per live cell per image of neurons in primary culture; 100 to 300 cells per image, 41 to 45 images per genotype. Live cell ROIs were created by masking out NucView 405+ (active caspase-3) cells from those of NeuroFluor NeuO+ cells (****P < 0.0001, two-way ANOVA, Tukey’s multiple comparisons test). Error bars in SEM.

To begin to understand how neuronal TMEM16F could affect tau pathology, we wanted to investigate how TMEM16F might affect neurons with phospho-tau burden. To do so, we used an in vitro neuronal model, where we could perform perturbations and analyses to cultured neurons with pathogenic tau burden, by testing primary hippocampal neurons from control 16F WT, 16F KO, PS19+ 16F WT, and PS19+ 16F KO mice. After 3 wk in cultures, PS19+ 16F WT neurons exhibited AT8+ staining, while control neurons did not (they only showed baseline background staining) (Fig. 4 *A* and *B*). Western blots with AT8 antibody monitoring of cell lysate confirmed that PS19− neurons lacked hyperphosphorylated tau (Fig. 4*C*). In this system, we found that while both PS19+ 16F WT and PS19+ 16F KO neurons developed hyperphosphorylated tau, there was no difference in the amount of AT8 signal in primary culture of neurons (Fig. 4*B*).

Neurons with phospho-tau burden from PS19 mice have previously been shown to aberrantly expose phosphatidylserine, which can lead to premature efferocytosis (49). While a different scramblase, XKR8, normally facilitates phosphatidylserine exposure as the “eat-me” signal on apoptotic cells (50), a recent study showed that exposure of phosphatidylserine on dorsal root ganglion neurons after cerebral ischemia is reduced with removal of TMEM16F (27). Hence, we wondered whether TMEM16F might play a role in exposing phosphatidylserine in neurons from PS19 mice.

To look at phosphatidylserine exposure in primary culture of living cells without fixation, we stained neurons with or without TMEM16F and with or without human P301S (PS19) mutant tau [days in vitro (DIV) 18 to 21] with a phosphatidylserine marker (annexin V) and a marker for live neurons (NeuroFluor NeuO). Because phosphatidylserine is also exposed on apoptotic cells, we additionally stained these neurons with an active caspase-3 reporter (NucView) (a reporter of apoptosis) and excluded all neurons that had any caspase-3 activity from our analyses. We confirmed that phosphatidylserine exposure in tauopathy PS19+ 16F WT neurons was greater than that of control PS19− 16F WT neurons (Fig. 4 *C* and *D*). We also found that KO of TMEM16F in PS19+ neurons reduced the level of exposed phosphatidylserine to that of control PS19− neurons (Fig. 4 *C* and *D*). Because the only difference in PS19+ neurons examined in this study was the presence or absence of TMEM16F, these data indicate that TMEM16F is facilitating exposure of phosphatidylserine, which may ultimately be affecting the pathology in tauopathy mice.

## Discussion

TMEM16F, with the dual function of calcium-activated lipid scramblase and ion channel, is broadly expressed throughout the body, but the study of TMEM16F functions in the nervous system has been limited. We found that removal of TMEM16F in the PS19 mouse model of tauopathy was protective at 6 to 7 mo of age, as indicated by the reduction of pathology in both the number of neurons with pathogenic hyperphosphorylated tau and the levels of microgliosis within the hippocampus (Figs. 1–3). Moreover, conditional KO of TMEM16F from neurons led to a reduction in pathology (Fig. 3), while conditional KO of TMEM16F from microglia did not result in detectable effects (Fig. 2). These observations indicate that neuronal TMEM16F is a driver of pathology observed at this time point.

We also found that TMEM16F mediated aberrant phosphatidylserine exposure in phospho-tau-burdened neurons (Fig. 4 *C* and *D*). How TMEM16F in neurons contributes to overall pathology is an intriguing open question for future studies, but this finding may provide a clue as to how tau burden can progress. Work from our lab has previously assessed the role of TMEM16F in the dynamics of EV formation. Han et al. demonstrated the importance of TMEM16F-dependent exposure of phosphatidylserine and Ca^2+^ influx in the formation of giant plasma membrane vesicles, a model for plasma membrane budding to form EVs (10). This is particularly interesting because one mechanism by which hyperphosphorylated tau oligomers and aggregates can spread is through EVs (37).

EVs are membrane-enclosed bodies of various origins and sizes that are released into the extracellular space to transport lipids, proteins, and various RNA species (51). Two main bioactive populations consist of 1) microvesicles (MVs) (~100 nm to 1 μm), also known as microparticles or ectosomes, which arise from direct outward budding of the plasma membrane and 2) exosomes (~30 to 100 nm), which are intraluminal vesicles (ILVs) that are released through exocytosis of multivesicular bodies (MVBs) (51).

Normal tau is known to associate with the plasma membrane (52, 53) and is present in MVs under physiological conditions and in exosomes when overexpressed (54). Studies have demonstrated that exosomes from rTg4510 mice (human tau P301L mutation) and brain-derived EVs from THY-tau30 mice (human tau P301S and G272V mutations) contain seed-competent tau and are capable of transmitting tau pathology in vivo (55, 56). However, these studies did not study whether MVs can also contain and transmit pathogenic tau. More sensitive assays are necessary to properly identify aggregated tau within these vesicle populations.

TMEM16F is known to play a critical role not only in exposing phosphatidylserine but also in the formation and release of MVs (19). Reduction of MVs by KO of TMEM16F affects blood coagulation (19), bone mineralization (57), viral particle shedding (58), and arthritis (22). The system developed by Han et al. helped to better understand how TMEM16F may be involved in releasing MVs by showing the requirement of phosphatidylserine exposure in the formation and release of plasma membrane-derived vesicles (10). Hence, our finding that neurons with phospho-tau burden have increased TMEM16F-mediated lipid scrambling (Fig. 4 *C* and *D*) raises the possibility that these pathological neurons may release MVs containing toxic tau oligomers through TMEM16F and further promote pathogenic tau spreading.

In our study, we found that at 6 to 7 mo of age, PS19 mice with removal of TMEM16F from neurons had fewer AT8+ CA1 pyramidal neurons (Figs. 1–3) while no difference was found in AT8+ signal from neurons in primary culture (Fig. 4 *A* and *B*). This difference might be due to a difference in the age of neurons and/or the presence of other cells and factors within the extracellular space in the brain that could aid in the propagation of phospho-tau in vivo in a TMEM16F-dependent manner, possibly through MVs. Our in vitro cultures utilize neurons from embryos, while our in vivo studies examine neurons from 6- to 7-mo-old mice. It is possible that hyperphosphorylated tau may have just appeared in cultured embryonic neurons, but not to the same extent of 6- to 7-mo-old mice. Other factors or cells in the brain that are absent in culture may also be contributing toward spreading of hyperphosphorylated tau. Several studies have demonstrated that astrocytes may be involved in transfer of pathogenic proteins (59, 60). The likely absence of these glial cells in our neuronal cultures could contribute to the difference between observed levels of AT8 in our in vitro and in vivo studies.

One technical limitation of this current study is that it is assessing early tau pathology. At 6 to 7 mo of age, PS19 mice do not have widespread neuronal loss, which occurs when these mice are 9 mo old (28). Brelstaff and colleagues suggest that phosphatidylserine exposure in phospho-tau-burdened neurons leads to microglial efferocytosis of living neurons in tauopathy and drives pathology (49). The exposure of this eat-me signal on neurons leads to their microglial engulfment and neuronal loss. Several studies implicating TMEM16F and microglial inflammatory states also use neuronal loss as a metric of interactions between microglia and neurons (23, 27). In 6- to 7-mo-old mice, however, phosphatidylserine exposure would not yet have led to efferocytosis. For these considerations, we do not exclude the possibility that microglial TMEM16F may also affect pathology at later time points. Further studies are necessary to examine whether and to what extent TMEM16F in microglia may affect pathology of PS19 mice.

In summary, our study revealed that removal of TMEM16F reduced tau pathology in 6- to 7-mo-old PS19 mice, a phenotype attributable to the removal of TMEM16F from neurons but not microglia. Our study of primary neuronal culture raises the possibility that TMEM16F-mediated exposure of phosphatidylserine on neurons with phospho-tau burden may contribute to the pathology. These findings provide the basis for future studies and possible therapeutic targeting of TMEM16F in tauopathy.

## Materials and Methods

### Animals.

TMEM16F KO mice and TMEM16F flox/flox mice were generated as described previously and backcrossed to C57BL/6J background for two to four generations (18). For complete KO mice, mice were maintained through F1 C57BL/6J/129S1 het × het pairings, as further backcrossing results in lethality of KO mice likely due to TMEM16F involvement in placenta formation (61). TMEM16F flox/flox mice were bred with either Cx3cr1-Cre (Jackson laboratory, stock #025524) or Baf53b-Cre (Jackson laboratory, stock #027826) mice. P301S tau (PS19) male breeders were purchased from Jackson laboratory (stock #008169) and crossed to TMEM16F KO mice, Cx3cr1-Cre flox/flox mice, or Baf53b-Cre flox/flox mice. PS19 mice show great heterogeneity in disease progression and pathology, but males exhibit more consistent phenotypes. Therefore, for this study, we used male littermates from one to two litters per six to ten breeding pairs.

All animal procedures were approved by the UCSF Institutional Animal Care and Use Committee and performed according to the guidelines provided.

### DAB Immunohistochemistry.

Immunohistochemistry is as previously described (62). Free-floating sections were first washed in 1x Tris buffered saline (TBS). Sections were quenched with 1% hydrogen peroxide (H_2_O_2_) in TBS and washed in TBS. Blocking was combined with primary incubation in TBS++++ media (1x TBS, 0.13 M glycine, 1.5% bovine serum albumin, 0.4% Triton X-100) at 300 µL per 24 well dish for 3 h with shaking, at room temperature. Either AT8 (1:500, mouse) (Invitrogen, #MN1020) or Iba1 (1:3,000, rabbit) (Wako Chemicals, #019-19741) antibodies were used. After incubation, sections were washed with TBS and incubated with 1:300 secondary HRP anti-mouse or anti-rabbit (Invitrogen) antibody in TBS++++ for 1 h with shaking, at room temperature. ABC-HRP peroxidase (Vector Laboratories) was prepared, as instructed (1:300 A + 1:300 B) in TBS++++. Sections were washed with TBS and replaced with ABC solution for 1 h with shaking, at room temperature. Afterward, sections were washed in 0.1 M Tris pH 8.0 and then stained with DAB for either 1 min (AT8) or 5 min (Iba1) with constant shaking. Staining was performed in small batches to control the time within solution as close as possible to either 1 or 5 min, within 2 or 3 s. DAB solution was prepared fresh before development (0.1 M Tris pH8.0, 1x DAB, 1.5% H_2_O_2_). Finally, sections were washed with 0.1 M Tris and mounted onto coverslips and allowed to dry fully overnight. Slides were washed in distilled water (dH_2_O) with shaking and then in FD cresyl violet 1:1 solution for 5 min. Slides were washed in dH_2_O and then went through a series of dehydrations. First, slides were submerged in 95% ethanol (EtOH) with 0.1% glacial acetic acid for 15 s, then incubated in two 100% EtOH washes for 2 min each. Slides were then incubated two times in xylene for 5 min each and finally coverslipped using Permount mounting media (Fisher). After drying, sections were imaged using an Aperio AT2 slide scanner (Leica Biosystems, Buffalo Grove, IL). Images were processed and analyzed with Slide Scope Virtual Scan, ImageJ software, and Python scikit and napari plugins.

Genotypes were blinded to the researcher performing each analysis. Before each analysis, ROIs were traced in ImageJ.

For AT8+ quantifications, strongly labeled AT8+ neurons within the pyramidal layer of CA1a-b were counted. Traced ImageJ ROIs were extracted and color deconvoluted into cresyl violet and DAB channels using the scikit-image package in Python (63). Cells were then segmented using Voronoi-Otsu labeling with the scikit-image plugin napari_segment_blobs_and_things_with_membranes on cresyl violet images (64). A predetermined threshold (established using TMEM16F KO mouse samples) was applied to DAB images and cells that contained at least 50% AT8+ signal were counted. Three to four sections per sample, around Bregma −1.8, were quantified and averaged. This threshold was applied to all genotypes, and the same criteria of counting were used for all cohorts. Data are presented as number of AT8+ neurons normalized by area of interest and total number of neurons per area (% AT8+ neurons, neuronal density in the CA1 pyramidal layer).

For Iba1+ quantifications, three ROIs [CA1a-b, (CA1c, CA2, and CA3), and DG] were traced for one section per sample in ImageJ. Particle analysis was performed using the ImageJ plugin on images with a predetermined threshold for each experiment. Microglial counts were normalized to the area (mm^2^) of traced ROIs.

### Primary Neuronal Cultures.

For primary hippocampal and cortical neuronal cultures, cortices and hippocampi were dissected from E15-E18 pups from PS19− 16 WT × PS19+ 16F WT or PS19− 16F KO × PS19+ 16F KO mouse breeding pairs. During dissection, while tissue from each pup was collected individually in dissection medium (HBSS without Ca^2+^ and Mg^2+^, 50 U/mL PenStrep, 1 mM sodium pyruvate, 20 mM HEPES, 0.45% glucose), pups were genotyped and samples were pooled based on genotype prior to papain digestion. Dissection medium was replaced with dissection medium with 200 U/mL papain (that had been heat-activated for at least 30 min at 37 °C) for 20 min at 37 °C. Papain medium was then removed, and cells were washed with plating medium [Dulbecco’s Modified Eagle’s Medium (DMEM) with high glucose (Gibco), 10% FBS, 50 U/mL PenStrep]. Neurons were triturated with three to four subsequent flamed glass pipette trituration steps. During each step, after tissue settled, supernatant was passed through a 70 µm filter. Neurons were counted, resuspended in plating medium, and plated onto 0.01% poly-L-ornithine (Sigma) coated, HNO_3_-treated coverslips at 60,000 cells per 12 mm glass coverslip or 1 mg/mL poly-L-lysine (Sigma) coated plastic dishes (320,000 cells per 12-well or 4.5 million cells per 10 cm dish). After 30 min at 37 °C, plating medium was removed and replaced with maintenance medium (Neurobasal-Plus, 50 U/mL PenStrep, 500 µm Glutamax, 2% B27-Plus). On 3 DIV, 1.66 µM floxuridine (FdU) was added and every 3 to 4 d thereafter, half of the medium was replaced with maintenance medium.

### Immunocytochemistry.

Cells on coverslips were washed three times with PBS and then fixed for 8 min at room temperature with 4% PFA in PBS. Cells were washed three times with TBS and incubated in blocking/primary solution TBS++++ for 3 h at room temperature or overnight at 4 °C. MAP2 (1:100, rb) (Chemicon, #AB5622) and AT8 (1:500, ms) antibodies were used. Cells were washed two times with TBS and two times with TBSTx (TBS, 0.1% Triton X-100). Coverslips were then incubated for 1 h with secondary 1:500 Alexa 555 or Alexa 488 anti- mouse or rabbit antibody (Invitrogen). They were washed two times with TBS and two times with TBSTx. Coverslips were finally mounted onto coverslips using DAPI FluorMount-G (SouthernBiotech). Once dry, coverslips were imaged using an SP8-X inverted confocal microscope with HyD hybrid detectors (Leica Microsystems, Wetzlar, Germany).

### Biochemistry.

Biochemistry is as described previously (62). Cell pellets were lysed using RIPA buffer (ThermoFisher, #89901). The protein content of resuspended cells was quantified using Pierce BCA protein assay kit (ThermoFisher, #23227) captured on a Synergy H4 plate reader (BioTek Instruments, Winooski, VT) and SDS sample loading buffer was added to samples. SDS-Page gels were loaded with 20 µg protein per well, run, and then transferred to nitrocellulose membrane using wet transfer with a Mini Blot module transfer system (ThermoFisher). After transfer, blots were washed three times quickly with H_2_O, then blocked in SuperBlock Blocking Buffer (ThermoFisher, #37515) at room temperature, shaking. After three quick H_2_O washes, blots were cut at around 42 kDa and each blot was incubated in primary antibody in TBST overnight at 4 °C. After primary incubation and three quick H_2_O washes, blots were washed twice with H_2_O and then one time with TBST. Antibodies used were AT8 (1:500, mouse) and GAPDH (1:15,000, mouse). Washed blots were then incubated with 1:25,000 secondary HRP anti-mouse antibody (Invitrogen) for 1 h at room temperature under shaking. Finally, after three quick H_2_O washes, they were washed two times with H_2_O, one time with TBST, and then processed with either West PicoPlus or Femto ECL for 3 min (Thermo Fisher) and scanned on a C-DiGit Blot Scanner (LI-COR, Lincoln, NE).

### Phosphatidylserine Exposure Assay.

Phosphatidylserine exposure assay was performed as described previously (62). On DIV 16 to 18, cultured primary neurons were washed twice with DPBS and then incubated with 2 µM NeuroFluor NeuO (Stem Cell Technologies, #01801) in neuronal maintenance media for 1 h at 37 °C. Neurons were washed two times with maintenance media and left until stained or three times with physiological saline solution (140 mM NaCl, 4 mM KCl, 2.5 mM CaCl_2_, 1 mM MgCl_2_, 10 mM dextrose, 10 mM HEPES, 0.1 mM EGTA, pH 7.4) (PSS) if they were about to be processed. Neurons were incubated in Annexin V 647 (1:200) (ThermoFisher, #A23204) and NucView (1:200) (Biotium, #10405) for 15 min at 37 °C. Neurons were washed three times with PSS and then imaged using a Leica SP8-X inverted confocal microscope with HyD hybrid detectors, adaptive focus control, and Okolab environmental control incubator cage (37 °C, 5% CO_2_). Then, 40 to 45 areas of the coverslip were selected based on NeuroFluor NeuO signal and then imaged.

Image analysis was performed on ImageJ. A predetermined threshold for NeuO+ signal was set per experiment to segment and create ROIs for neuronal cell bodies. NucView signal (active caspase-3 activity) was measured within each ROI and any ROI (cell body) with signal was masked out and removed from analysis. Neuronal cell bodies absent of NucView signal were measured for annexin V intensity and annexin V intensity per cell body was averaged per image.

### Statistical Analysis.

Datasets were first tested for normality using the Shapiro–Wilk test. Depending on this result, then either Welch’s *t* test or two-way ANOVA with Tukey’s multiple comparison test (normal) or Mann–Whitney (nonparametric) was performed to test for significance.

## Supplementary Material

Appendix 01 (PDF)

## Data Availability

All study data are included in the article and/or *SI Appendix*.
