# Supplementary material for: TMEM16F exacerbates tau pathology and mediates phosphatidylserine exposure in phospho-tau-burdened neurons
Source: Proc Natl Acad Sci U S A. 2024 Jun 28;121(27):e2311831121. doi: 10.1073/pnas.2311831121 (PMC11228522; doi:10.1073/pnas.2311831121)
Supplement: Supplementary file 1 — Appendix 01 (PDF) [file pnas.2311831121.sapp.pdf]

PNAS

Supporting information for

TMEM16F exacerbates tau pathology and mediates phosphatidylserine exposure in phospho-tau-burdened neurons

Mario V. Zubia[a,b,c,d], Adeline J.H. Yong[b,c,d], Kristen M. Holtz[e], Eric J. Huang[a,f], Yuh Nung Jan[b,c,d], Lily Y. Jan[b,c,d,1]

[a] *Biomedical Sciences Graduate Program, University of California, San Francisco, CA 94143;*
[b] *Department of Physiology, University of California, San Francisco, CA 94143;*
[c] *Department of Biochemistry and Biophysics, University of California, San Francisco, CA 94143;*
[d] *Howard Hughes Medical Institute, University of California, San Francisco, CA 94143;*
[e] *Department of Autonomy, Skydio, San Mateo, CA 94402;*
[f] *Department of Pathology, University of California, San Francisco, CA 94143;*

[1]To whom correspondence may be addressed.
Email: lily.jan@ucsf.edu

This PDF file includes:

Figures S1 to S2

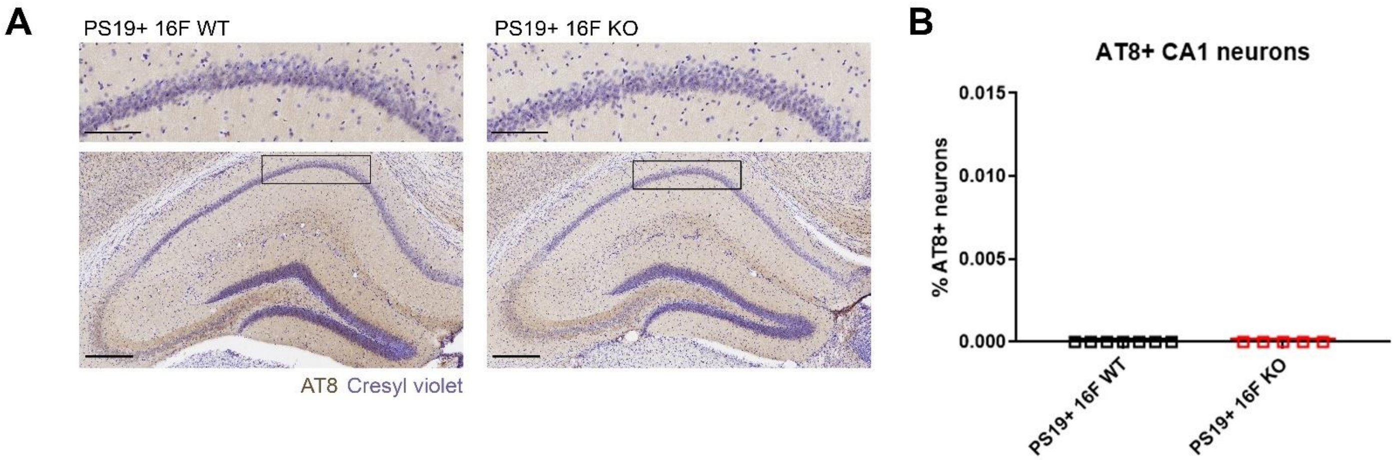

Figure S1. Hyperphosphorylated tau pathology was not detectable in 3-month-old TMEM16F knockout mice.

(A) Representative images of AT8 immunostaining of hyperphosphorylated tau in the hippocampus and CA1 (*Inset*) in 3-mo-old PS19+ TMEM16F WT (16F WT) and TMEM16F KO (16F KO) mice. Cresyl violet, neuronal counterstain. (Scale bar, 300 µm for hippocampus and 100 µm for *Inset*.)

(B) Quantification of AT8+ neurons within CA1 pyramidal layer in PS19+ WT, n = 7 and PS19+ KO mice, n = 5; 1 hippocampal section/mouse.

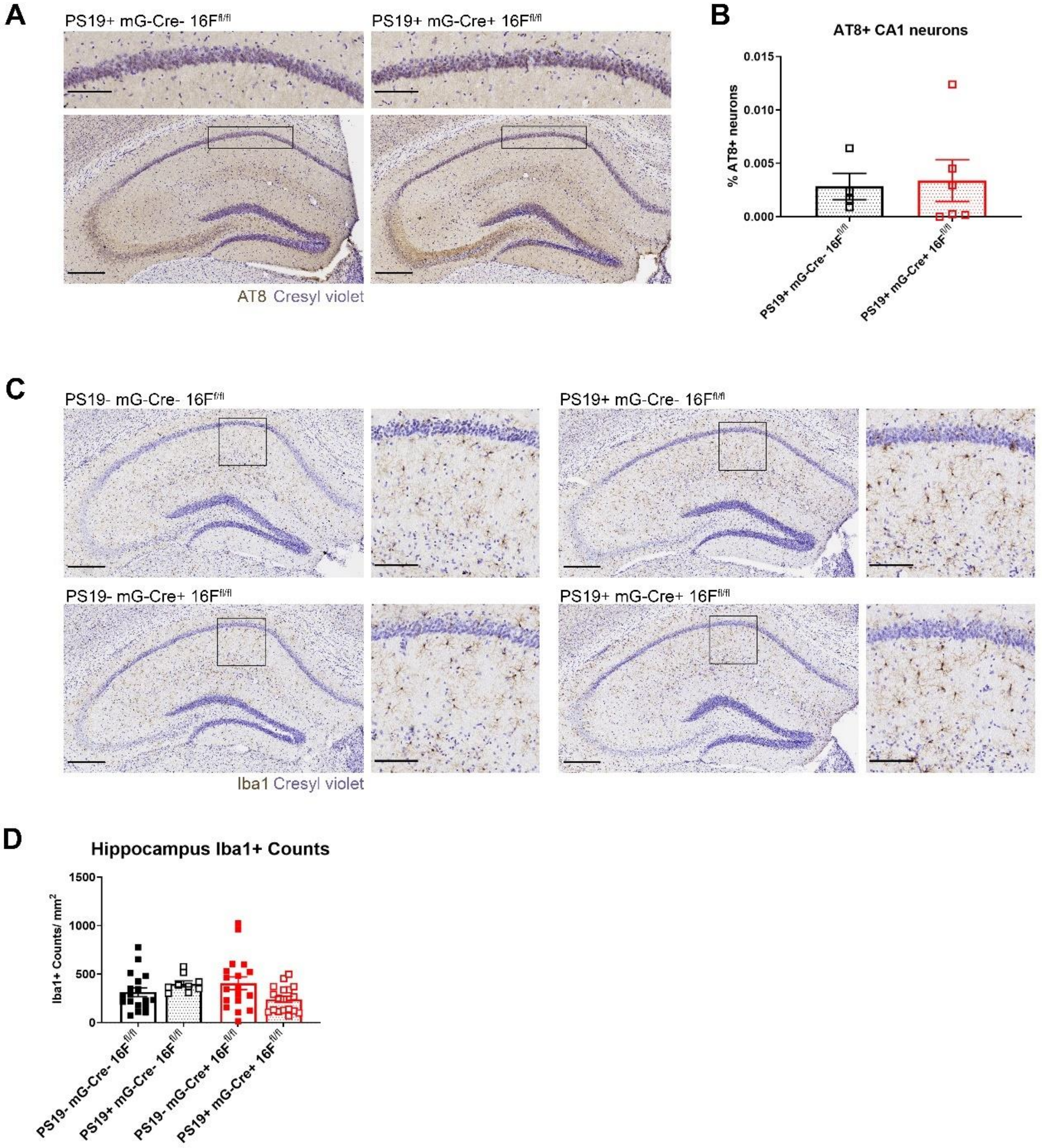

Figure S2. 6-month-old microglial conditional TMEM16F knockout mice without detectable pathology.
(A) Representative images of AT8 immunostaining of hyperphosphorylated tau in the hippocampus and CA1 (*Inset*) in 6-mo-old PS19+ microglial TMEM16F WT (mG-Cre- 16F$^{fl/fl}$) and microglial TMEM16F conditional knockout mice (mG-Cre+ 16F$^{fl/fl}$). Cresyl violet, neuronal counterstain. (Scale bar, 300 µm for hippocampus and 100 µm for *Inset*.)

(B) Quantification of AT8+ neurons within CA1 pyramidal layer in PS19+ mG-Cre- 16F$^{fl/fl}$, n = 3 and PS19+ mG-Cre+ 16F$^{fl/fl}$ mice, n = 3; 3 to 4 hippocampal sections/mouse. (*P* = 0.7619, Mann-Whitney test). Error bars in SEM.

(C) Representative images of Iba1 immunostaining of microglia in the hippocampus and CA1 (*Inset*) of 6-mo-old PS19- mG-Cre- 16F$^{fl/fl}$, PS19- mG-Cre+ 16F$^{fl/fl}$, PS19+ mG-Cre- 16F$^{fl/fl}$, and PS19+ mG-Cre+ 16F$^{fl/fl}$ mice. Cresyl violet, neuronal counterstain. (Scale bar, 300 µm for hippocampus and 100 µm for *Inset*.)

(D) Quantification of number of microglia per area (count/mm^2) in the hippocampus of 6-mo-old PS19- mG-Cre- 16F$^{fl/fl}$, PS19- mG-Cre+ 16F$^{fl/fl}$, PS19+ mG-Cre- 16F$^{fl/fl}$, and PS19+ mG-Cre+ 16F$^{fl/fl}$ mice, n = 4 – 5 per genotype, 3 regions of interest per mouse. (ns = $P > 0.05$, two-way ANOVA, Tukey's multiple comparisons test). Error bars in SEM.
